# Functioning Changes in Varying Ways After Retirement: A Scoping Review

**DOI:** 10.1177/00469580221142477

**Published:** 2023-01-05

**Authors:** Pauliina Saha, Jatta Salmela, Tea Lallukka, Anna Liisa Aho

**Affiliations:** 1 Tampere University, Tampere, Finland; 2 University of Helsinki, Helsinki, Finland

**Keywords:** Retirement, functioning, scoping review, retired, aging, public health

## Abstract

The association between retirement and functioning remains still poorly known. This scoping review examines physical, social, cognitive, and mental functioning after retirement, describes the changes in them, determines the different aspects that affect functioning, and documents the main characteristics of the phenomenon. We systematically scoped the relevant studies on functioning after retirement using CINAHL, MEDLINE, Medic, and PubMed databases. This scoping review included both qualitative and quantitative studies. The studies were analysed with inductive content analysis. After retirement, functioning was found to decline but also improve, and additionally, inequalities in functioning emerged. Functioning after retirement changed in ways which were: declining functioning, improving functioning, and inequalities in functioning. Only a few qualitative studies were found. This scoping review shows that functioning after retirement changes in varying ways. The results show that more qualitative research is needed to help us gain a more profound understanding on, for example, individuals’ motives to improve leisure, physical, and social activities after retirement, which are likely to contribute to changes in functioning. Additionally, further longitudinal studies would offer knowledge about the long-term effects of retirement on the different dimensions of functioning.

**What do we already know about this topic?**
The associations between different domains of functioning and retirement remain somewhat unknown, and the available evidence is conflicting.
**How does your research contribute to the field?**
The results of this scoping review showed that functioning changes in varying ways after retirement depending on the dimension of functioning.
**What are your research’s implications toward theory, practice, or policy?**
This study underlines the need for further research around the field to gain a more profound understanding about retirement at individual level.

## Introduction

The 21st century is predicted to become a time of population aging, given that the proportion of aged people increases in all countries, although with different intensities.^1^ Population aging has and will continue to increase the number of retirees, and the proportion of society they represent. This demographic trend will radically change the whole society, and the changes will be seen in national and international economics and politics, the organisations where the people currently work, and in the impact on the well-being of following generatiwons. However, with a better insight of retirement, it is possible to understand, predict, and plan for these potential changes in society.^2^

Good health and functioning forms the base for an independent, balanced, and satisfied life after retirement.^3^ Healthy aging is a process of developing and maintaining the functional ability that enables wellbeing in older age.^4^ Peoples’ physical, psychosocial, and social capacity to cope with daily activities in the environment they live in can be identified with the concept of functioning. The positive and negative impacts of the environment can either improve or deteriorate functioning.^5^ As a concept, functioning can have several dimensions. This review considers the following dimensions: physical, mental, cognitive, and social functioning.^5^ Physical functioning means a person’s ability to cope with daily activities. Cognitive functioning refers to a co-functioning of different information processing areas that makes it possible to cope in everyday life. Social functioning can mean the ability to act in close relationships and communities. Mental functioning consists of, for example, mental resources, opportunities to influence one’s life, the existence of satisfying social relationships, and positive perception of oneself.^6^ These different dimensions are interconnected with the requirements and qualifications of the person’s environment, and individuals’ health and other qualities.^5^ This review focuses on both the negative and positive changes in functioning after retirement.

Retirement is a life-course transition which occurs in late adult life.^7^ Retirement can be approached in several ways, and can affect or be affected by variables at the larger level of society, the employing organisations from which a person retires, and the individual retirees themselves. We focus on the individual retirees themselves. At this level, the main interest is understanding the individual qualities and current circumstances that might influence their retirement, such as demographic factors, abilities, and health. Also, situational circumstances, such as an individual’s type of job and their family situation, might affect retirement. Another main interest is toward how the individuals themselves are impacted by their retirement: what happens immediately after retirement, and which effects retirement has on the individual years after they have retired.^2^ In most developed countries, the social security programs are financed as they occur (pay-as-you-go basis).^8^ This amplifies a lifetime income inequality between people. Those who have received a larger salary, also usually receive a higher pension. They possibly draw this higher pension for longer due to their greater life expectancy.^9^ This review examines the socioeconomic inequalities related to retirement.

The associations between different domains of functioning and retirement remain somewhat unknown, and the available evidence is conflicting. For instance, reviews and meta-analyses studying the association between cognitive functioning and retirement suggest that while retirement may not have a negative impact on adults’ global cognition, it displays slightly adverse influences on memory-related skills.^10^ While examining age-related cognitive decline, the results diverge when examining fluid cognitive abilities, and retirement appears to accelerate the rate of cognitive decline in preserved abilities.^11^ A systematic review about retirement and physical activity concluded that exercise and leisure-time physical activity increased after the transition into retirement, however, the total amount of physical activity among retirees was not determined.^12^

Scoping reviews are useful for examining the emerging evidence in the case that it is still obscure what other, more specific questions can be posed and valuably addressed by a more explicit systematic review.^13^ The purpose of this scoping review was to investigate functioning and its changes after retirement, and determine the different aspects that affect functioning. More specifically, the aim was to gain knowledge about a person’s functioning outcomes after retirement, to identify the studies available, and the possible knowledge gaps that exist in the field. This will help form more specific research questions for systematic reviews, and help to specify what type of studies are needed to investigate further how different dimensions of functioning changes after retirement. Collectively, this information could be used in developing actions to prevent deterioration in a person’s functioning after retirement.

## Methods

### Search Strategy and Study Selection

The review was conducted as a scoping review to determine the scope or coverage of a body of literature and the studies available in a chosen field.^13,14^ In terms of analysis, there is a certain relevance given to the time, location, source, and origin of the literature chosen while conducting the review.^14^

In scoping reviews, the first planning phase starts with a formulation of the main research question.^15^ The research question was “How does functioning change after retirement?” This was formed based on the PCC (Population, Concept, and Context) model, which is suitable when scoping what kinds of studies exist related to the subject of interest.^16,17^ After this, the planning phase continued with defining the inclusion and exclusion criteria. Population (P) included people in retirement, and the concept (C) was functioning (including physical, social, psychosocial, and cognitive functioning) after retirement. Context (C) included life after retirement, functioning after retirement, studies conducted in developed Western countries, and published in 2011 to 2021. This criterion was made since the demography of aging develops parallelly in these countries, but not necessarily with the same magnitude in developing countries.^18^ The type of studies for inclusion were peer-reviewed studies written in English. The criteria followed the research question and were formed before conducting the review.^15^

**Figure 1.**
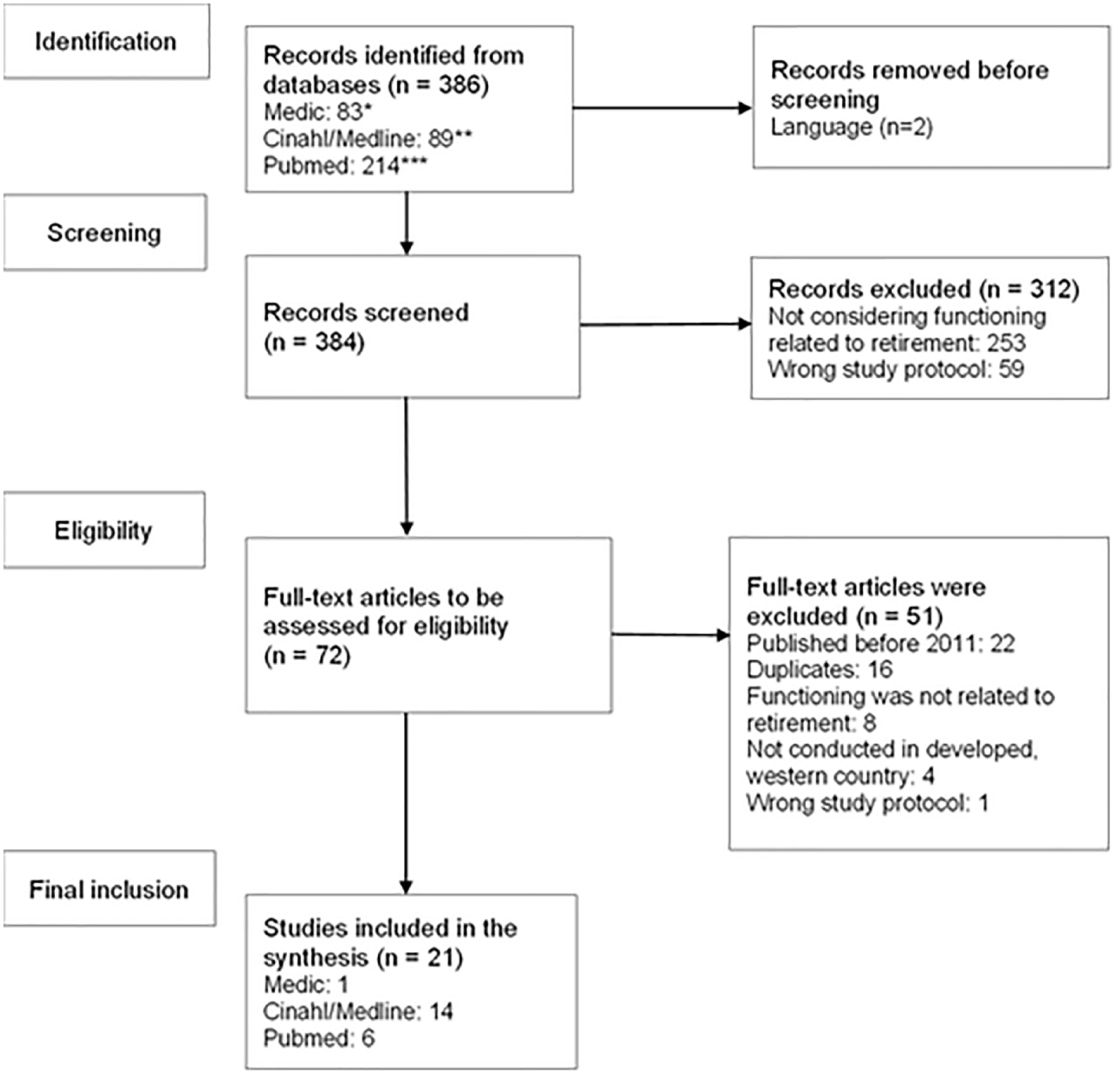
PRISMA flow chart of the literature search. *Keywords: (“International Classification of Functioning, Disability and Health” (keyword) OR “Physical Functional Performance” (keyword)). **(Functioning “[Title/Abstract] OR “health function*” [Title/Abstract] OR “physical functioning” [Title/Abstract] OR “psychosocial functioning” [Title/Abstract] OR “social functioning” [Title/Abstract] AND “retire” [Title/Abstract] AND “after” [Title/Abstract]. ***(MH “retirement”) AND (AB after* OR TI after*) AND ((TI (MH “International Classification of Functioning, Disability and Health”) OR (MH “Functional Assessment”)) ((AB ((MH “International Classification of Disability and Health”) OR (MH Functional Status) OR (MH “Functional Assessment”) OR (TI functioning* OR AB functioning*).

The literature search was conducted in March 2021 in 4 different databases, CINAHL, MEDLINE, Medic, and PubMed (Figure 1). Pubmed, MEDLINE, Medic, and CINAHL are all major databases which consider the topics related to the research question, such as allied health and medical literature. This amount of databases seemed suitable since the use of more databases did not remarkably expand the amount of suitable search results. The chosen keywords were formed from the same base including keywords of “functioning” and “retirement,” and featured in the PCC model and the research question.^19^ The whole literature search process is described in the supplementary file.

### Analysis

The literature of the scoping review was analysed systematically based on inductive content analysis. Content analysis is one of the most used methods for analysis in qualitative research.^20^ When the purpose of a scoping review is to identify or clarify concepts and definitions, descriptive qualitative techniques such as descriptive qualitative content analysis including a basic coding of data can be a useful approach.^16,21^ When applied in a review, content analysis is used as a way to organise the literature, and not as a particular tool for analysis. Generally, this follows a process of 3 phases: reducing the material, clustering the material, and abstracting the material.^21^ This analysis was conducted by 1 researcher.

According to Kyngäs et al,^21^ data was properly identified before starting the content analysis. The analysis started with reading the data profoundly. The chosen studies were read first at the phase when choosing the suitable studies for the analysis. The reading phase continued by reading all the chosen studies to gain an overview of the data. Then reading continued with making highlights and notes while reading the studies. These highlights and notes made the base for continuing to the next phase of the analysis. The analysis continued with choosing the units for analysis. The units for analysis were chosen to answer the research question and tabulated into an Excel table. In the analysis a unit meant 1 sentence including 1 meaning answering the research question. The chosen units formed the reductions (n = 218) that maintained relevant information. Reduction here meant summarising the sentence (ie, the unit) so that the meaning of the sentence didn’t change. This part was conducted by 1 researcher. The reductions were then analysed to find similarities and differences. This formed the base for the next step of forming categories. The data and the researcher’s interpretation allowed the reductions to be combined to form a named category. The analysis continued with combining the different subcategories into generic categories. At first, the reductions were divided into subcategories (n = 45), and were then divided further into generic categories (n = 11). Final synthesis formed the main categories (n = 3). The quality of the studies was assessed with JBI checklists for Qualitative Research (n = 1), Analytical Cross Sectional Studies (n = 2), Cohort Studies (n = 17), and Quasi-Experimental Studies (n = 1). These checklists include from 8 to 11 questions which are answered either “yes,” “no,” “unclear,” or “not applicable.”^15^ The quality assessment did not cause any exclusions. The quality assessment scores can be found from the Supplemental File (see Supplemental Table 1).

## Results

### Review Characteristics

The literature search returned 386 results. The results were evaluated based on title and abstract, searching for studies that would answer the research question, and which met the inclusion criteria. During this phase, 59 results were excluded based on the study protocol. The excluded results included, for example, non-scientific articles in trade journals or cohort profile descriptions, and some irrelevant systematic reviews that did not consider functioning after retirement. Overall, more than half of the results (n = 253) did not consider functioning related to retirement, and examined issues such as financial planning related to retirement, functioning after a certain disease state, and returning to work after sickness absence. Two results were excluded as the language was other than English. Altogether, 72 studies were included based on their title and abstract.

The transferred studies were evaluated, and a time restriction was set where 22 studies published before 2011 were excluded. The returns also included 16 duplicates which were excluded. The process continued with a more intense evaluation of the studies based on their full texts.^19^ At this stage, 8 studies were excluded since they did not answer the research question, and the functioning was not related to retirement. These excluded studies considered the effects of working after retirement or later in life (n = 4), functioning related to work life (n = 2), functioning in old age but not related to retirement (n = 1), and retirement planning (n = 1). This scoping review focused on studies conducted in Western countries, hence, 4 studies were excluded. One further study was excluded as it was a study protocol.

The final literature consisted of 21 studies (Figure 1). This included 17 quantitative, longitudinal studies.^22-38^ The rest of the quantitative studies included cross-sectional studies^39,40^ and 1 quasi-experimental study.^41^ One qualitative phenomenological study was included.^42^ Many of the studies were based on the same longitudinal data. Consequently, the chosen studies were based on 9 different cohort studies and 1 phenomenological study. Geographically, the studies were conducted in the USA,^22-24,35-37^ United Kingdom,^29-33,40,41^ Finland,^25-27,34^ Sweden,^28^ Norway,^42^ France,^39^ and the Netherlands.^38^ One of the studies considered only people on disability retirement.^22^ All the chosen studies are tabulated in the Supplementary File (Table 1).

**Figure 2.**
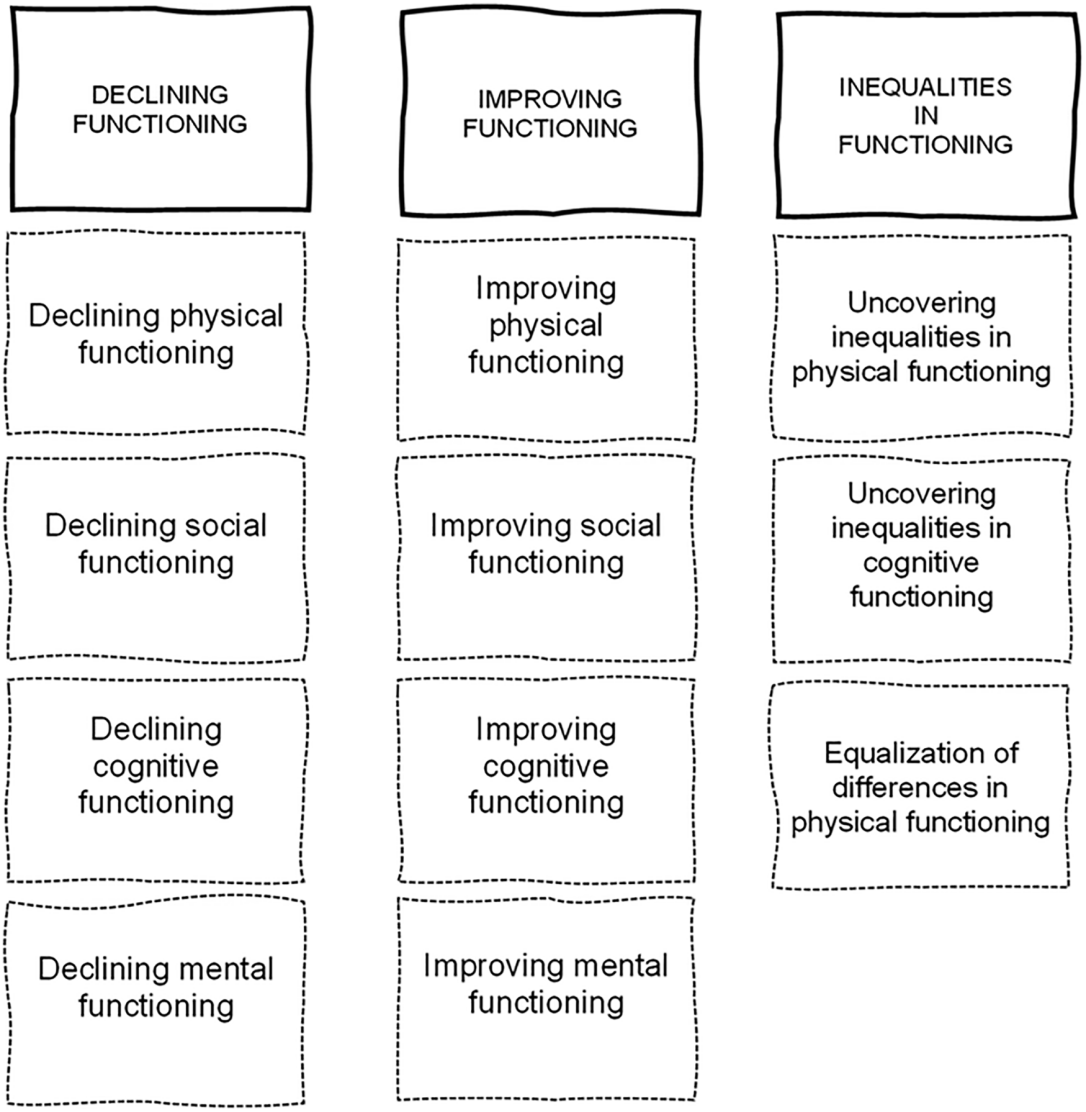
The changes in functioning after retirement. *Source.* Elaborated by the authors.

The age distribution was 18 to 101 years. Even though the focus was on retired people, some studies also included control groups which widened the age distribution to lower ages. The studies were published during 2011 to 2021. Most of the studies (67%) did not include explicit information of the participants’ years on retirement. Three of the studies^32,33,39^ reported the mean of how many years the participants had been retired. In this study the mean from these 3 studies is 10.7 years. Four of the studies^29,36,38,42^ reported the variation of how many years the participants had been retired. In this study the variation from these 4 studies is 0 to 15 years. After retirement, the changes in functioning included declining functioning, improving functioning, and inequalities in functioning (Figure 2).

### Declining Functioning

Declining functioning included declining physical, social, cognitive, and mental functioning (Figure S1). Declining physical functioning was associated with age and work-related characteristics such as high job strain, passive work, and occupational environmental hazards. Declining physical functioning was also associated with health and retirement itself. Age along with retirement was found to decline *physical functioning*,^25,26,34,35^ and difficulties and limitations in physical functioning increased with age.^34,35^ Also, the decreases in physical activity^26^ and a deterioration in physical functioning^25,34^ appeared to increase more steeply during retirement compared to being employed. Job features^27,32^ such as passive work^27^ were associated with a deterioration of physical functioning. High job strain,^28,33,36^ exposure to different types of workloads^27,32^ and imbalance in the effort-reward system^36^ were also associated with declining physical functioning. Additionally, different types of health factors, such as chronic diseases and a greater amount of lifestyle-related risks, were found to be additive for deterioration in physical functioning.^34^ A deterioration was found in perceived^41^ and measured^25,30^ physical health. Retirement itself was found to decline physical functioning,^25,30,34,41^ and reduced activity to participate in sports clubs, sports, and exercise activity.^41^

Retirement itself was further found to decline social functioning.^41,42^ Retirement caused confusion and negative thoughts related to the new situation after working,^42^ and the number of acquaintances met and the level of participation in social activities lowered after retirement.^41^ *Declining social functioning* was also found to be related to a lower cognitive level job.^40^

Retirement correlated with a decline in cognitive functioning,^22,23,30,38^ and was revealed in several tests measuring a person’s cognitive performance.^30,38^ The association between the retirement duration and declining working memory was found,^23^ but also age alone was found to be related with declining cognitive functioning.^28^ *Declining cognitive functioning* was associated with socioeconomic position. Especially, higher education was found to be related to a more rapid decline in cognitive functioning, yet, worse wealth and education at retirement were associated with a more rapid cognitive aging over time.^23^ Work-related factors were associated with declining cognitive functioning, such as moderate or high job demands,^31,33^ high job strain,^28,33^ low job control,^33^ and low complexity job.^22^ Additionally, health status and social isolation were associated with declining cognitive functioning.^23,31^

Retirement itself was associated with a subsequent decline in mental functioning.^29^ Age with relation to retirement was one of these factors,^29^ and an older age and more years of retirement were related to a poorer quality of life. Poor quality of life was also related to social isolation.^29^ Finally, *declining mental functioning* was found to have an impact on mental wellbeing,^41^ causing a lower lust of life and constant worrying about one’s health, for example.

### Improving Functioning

Retirement itself was found to be associated with *improving physical functioning*.^25,30,35,40,41^ The number of physical problems lowered,^41^ and better self-related health was reported.^42^ Increases in physical limitations lowered^35^ and measured physical functioning improved.^25^ Earlier retirement was associated with improving physical functioning because of a higher number of leisure-time activities.^40^ One of the factors in improving physical functioning was that retired people tend to preserve the physical activities that they have already established before retirement.^42^ Improving physical functioning was related to self-rated health,^42^ socioeconomic position,^29^ and workload.^27^ A higher occupational grade was associated with improving physical functioning. Also, a high exposure to physical workload and lower exposure to computer work were associated with improving physical functioning after retirement.^27^

Retirement itself improved social functioning,^30^ since retirees were involved in more leisure-time activities. A relation between work and social functioning was also found,^39^ and the more retirees reported that they liked the way work used to organise their life, the higher their social performance was after retirement. *Improving social functioning* was related to changes following retirement. Increased autonomy and a new perception of time affected retirees’ social functioning in positive ways. Retirees enjoyed having more time, and also the feeling that resulted from increased autonomy. This led to getting interested in making new relationships and engaging in social activities. However, the awareness of one’s role in existing relationships grew as well.^42^ After retirement, time was not considered as something that needed to be controlled, but rather something that was on the retiree’s side. This new perception of time led retirees to be more present in the moment, and this helped them to live according to their own priorities and to form a coherent narrative connecting one’s past, present, and future. This was concerned with having a footprint in the world, which could remain even after their death.^42^

Several means of improving cognitive functioning were found to occur after retirement.^23,24,28,29,31,33,39,40^ Retirement itself appeared to make the rate of change slightly less steep compared to the years leading up to retirement.^24^ Retirement timing was related to *improving cognitive functioning*, but not in a completely coherent way. Specifically, performance in different types of cognitive tests was significantly better with earlier retirement compared to statutory retirement. However, later retirement also showed better results in similar tests compared to statutory retirement.^29,40^ Work-related factors were associated with improving cognitive functioning in several ways. Favorable work characteristics contributed to improving cognitive functioning.^33,39,40^ An anti-push, positive consideration of former work keeping people in employment, was seen in greater cognitive functioning and in better performance in different types of cognitive performance tests.^39^ Also, an attachment to one’s job,^39^ higher job control,^33^ and low physical demands^40^ were related to improving cognitive functioning. High work-related mental demands were associated with improving cognitive performance,^24,31^ and with slower declines in memory.^24^ A higher job strain was related to better cognitive performance,^28,33,40^ and with a slower decline in cognitive performance.^28^ Previous leisure-time activities^39,40^ and new leisure-time activities after retirement^39^ were associated with improving cognitive functioning. Socioeconomic position was a key factor affecting cognitive functioning: both higher educational attainment^40^ and higher wealth^23^ were associated with better cognitive performance and less rapid declines in cognitive functioning over time. Retirement itself appeared to make the rate of change in cognitive functioning slightly less steep compared to the years leading up to retirement.^24^

Retirement itself was associated with *improving mental functioning*.^24,30,41^ Retirees had better mental health,^30^ and for men, the satisfaction with one’s health grew.^41^ A younger age at retirement^24^ was related to better mental health. For men, there was less worrying about one’s health, and men had in general better cognitive functioning.^41^ Higher occupational grade^29^ and higher educational level^24^ were both related to improving mental functioning. Better health, such as better self-rated health and fewer depressive symptoms, and work-related factors, such as having spent more years in the same occupation and higher mental job demands, were associated with improving mental health functioning.^24^

### Inequalities in Functioning

Retirement was associated with inequalities in functioning, in terms of age, sex, marital status, race, socioeconomic position, and job features (Figure S3).

*Inequalities in physical functioning* were found in terms of sex,^32,35^ race, marital status, and retirement age.^35^ Some physical limitations were more common among women than men.^32,35^ Furthermore, limitations were correlated with marital status,^35^ being more common among non-married/-partnered retirees. In regard to race, physical limitations were more common among races other than non-Hispanic whites.^35^ Additionally, retirement age was associated with limitations in physical functioning, limitations being more common among those retiring at an older age.^35^ Retirement also narrowed socioeconomic differences in physical functioning that were present before retirement.^35^ Notably, class inequalities were slightly narrower for retired individuals than for those in employment, and this narrowing after retirement continued while aging.

*Inequalities in cognitive functioning* were related to job characteristics,^22,24,31^ sex,^28^ and socioeconomic position.^38^ Cognitive functioning remained better among men than women.^28^ However, the feature of a greater job strain negatively affecting cognitive functioning was only significant for men.^28^ Those with more mental demands at work showed better cognitive functioning.^24^ This difference grew over time.^24^ High complexity jobs showed fewer consequences on cognitive function with people on disability retirement.^22^ Mental job demands had a stronger negative impact on socially isolated individuals compared to those who were non-isolated.^31^ Verbal memory levels remained higher among individuals with higher employment grades.^38^ Retirement was also shown to narrow socioeconomic differences in physical functioning that were present before retirement.^25^ Class inequalities were slightly narrower for retired than for employed. This narrowing at retirement seemed to continue toward the oldest ages.

## Discussion

This review sought to examine how functioning changes after retirement. The main results show that these changes are ambiguous. This review considered different dimensions within functioning, since functioning can be measured in multiple ways. The main dimensions of functioning addressed in this review and the theoretical framework employed were physical, social, cognitive, and mental functioning. It was revealed that functioning after retirement may change unpredictably, even within the same dimension of functioning. These results are in line with earlier reviews.^10,11,12^ This review showed that after retirement functioning can either decline or improve. Additionally, inequalities in functioning after retirement can be identified.

The changes in functioning after retirement is a widely researched topic but, to the best of our knowledge, no review before has been published to summarise the changes in functioning after retirement, and considering all these 4 dimensions of functioning. This study summarises the variety and the differences in drivers that either improve or decline different dimensions of functioning. Even though the changes are ambiguous, there is still the possibility to target research, and policy and health actions on certain areas based on the knowledge that this study brought. Different retirement policies might also affect a person’s functionality. The heterogeneity of the results might be a cause of varying policies among different countries. This review considered only studies conducted in developed, western countries. The policies are more similar among chosen countries, which might even out the differences caused by variety among different retirement policies.

Declining functioning was reflected in all main dimensions of functioning. However, Meng et al^11^ have asserted that there is a huge knowledge gap in the associations between retirement and age-related cognitive decline, underlining the need for future research in this field and also in the mechanisms that lie behind these associations. Several factors, such as a lower lust for life, and a constant worrying about one’s health, were found to be associated with a decline in mental functioning.

Higher age and more years since retirement were found to correlate with a decline in physical, cognitive, and mental functioning. Retirement was shown to decline social functioning. These results show that targeted preventive health actions are needed to support people both during and after the retirement process. This support should continue after retirement since it was shown that declines in functioning appear to grow steeper toward higher age. This type of information can be used when planning and assessing the impacts that promote well-being in this area.^5^ For example targeted health check-ups for elderly people could be an effective way to prevent the possible collapse in persons functioning.

Declining mental functioning was related to social isolation. From this can be presumed that social isolation possibly comes partially as a result of retirement when a person misses the daily social activities perceived from the work community. We think this is a concern that should be noticed on an individual level, by employers, and also in social policy. For example, employers could seek to maintain contact with retired workers by for example inviting them to social events in the workplace. This would attach the previous workers to the work community even after they have retired from the work. To an employer sector this would provide a way to gain and maintain the quiet information that is usually lost when the person leaves the workplace.

Employers responsibility toward employees should be considered both after retirement and throughout their career. Declining functioning was not influenced only by retirement, and it appears that targeting preventive health actions only toward retired or aging people is not enough. Koponen et al^6^ state that it is possible to lower morbidity by improving working conditions. The need to invest in preventive health was justified with the finding that several health-related factors, such as chronic diseases and a greater amount of lifestyle-related risks, were related to declining functioning. Socioeconomic position was also a present issue while examining declining cognitive functioning. Higher education was related to a more rapid decline in cognitive function, and worse wealth and education were associated with more rapid cognitive aging over time. This is in-line with previous studies which have found an association between lower socioeconomic position and decreases in physical activity after retirement.^12^ This shows that neither high or low education entirely protects against declining cognitive functioning, but the preventive actions needed are not similar.

Retirement was associated with improving functioning, and the factors related to improved functioning were intriguingly very similar with those related to declining functioning. These factors provide good examples of the possible positive changes that may be used to support the retirement process, both after retirement and also during work-life. Especially supporting individuals’ activity and positive thoughts related to the new and changing situations after working-life would appear to have an important effect on their functioning.

Increasing leisure-time activities seemed to have a positive effect on functioning which is similar to previous studies,^10-12^ although it remains unclear how retirement affects total physical activity. One explanation for not being able to clarify this association is the imprecise physical activity measures used in different studies. Also, there is a need to better understand the underlying motives behind improving leisure-time activity, for example, by using qualitative studies. Furthermore, longitudinal studies would be needed to gain information on whether these improvements are maintained in the long term after retirement.^12^

While high job demands were found to be associated with improving functioning. This finding is in line with many studies which have shown that higher education is related to higher performance in cognitive tests, and thus, education might explain these results.^43,44^ This also supports the suggestion presented above, that improving working conditions can affect an individual’s health in many positive ways. Alvarez-Bueno et al^10^ state that both individual and job characteristics should be considered while planning strategies to promote a healthy transition to retirement. As an additional consideration, higher socioeconomic position, measured by occupational grade, educational level, and wealth, was associated with improving physical, cognitive, and mental functioning.^10^

This review uncovered a number of inequalities while examining the changes in functioning after retirement. However, these appeared to develop differently depending on age, sex, marital status, race, socioeconomic position, and job features. These are especially important knowledge to consider when deciding where and among which groups targeted support is particularly needed. Socioeconomic position was a central determinant while examining declines and improvements in functioning after retirement. The Finnish Institute for Health and Welfare (2021) has presented 8 justifications to reduce health and welfare inequalities in Finland. Health and welfare equality is a fundamental right for everyone.^5^ When we reduce health and welfare inequalities, we also improve public health, reduce costs, secure services, raise employment rates, secure civil peace, and take part in reducing a global problem.^5^ Therefore, these findings urge us to consider individual characteristics when planning a healthy transition to retirement.^10^ While in most developed countries, the social security programs are financed as they occur,^8^ it amplifies a lifetime income inequality between people. This is 1 statement that should be considered in policy making.

Lastly, a positive finding related to improved functioning was the equalisation of differences in functioning. Namely, retirement itself appeared to narrow socioeconomic differences in physical functioning, and this positive progress appeared to continue to the oldest ages. This would be a particularly interesting and important topic for future research, especially in relation to the key factors that lie behind this finding, and the characteristics of people in different socioeconomic groups related to functioning.

The review revealed the low number of qualitative studies in the scholarly corpus. Thus, more qualitative research is needed to help us gain a more profound understanding on, for example, individuals’ motives to improve leisure, physical, and social activities after retirement. Additionally, longitudinal studies would offer knowledge about the long-term effects of retirement on the different dimensions of functioning. The ambiguity in the results might be attributable to the differences between occupational groups. Hence, specific interventions to different occupational groups such as lower and higher grade employees would be needed. Also qualitative studies would increase our understanding about the associations, and how different employees perceive the questions.

The use of similar measures between different studies would provide a way to synthesise these results and gain long-term data around the subject. Our results stated the major contribution of socioeconomic position to changes in functioning. One suggestion is to perform studies that distinguish between employees of low and high education. Another suggestion for researchers is to focus on the covariates behind the changes in function after retirement: for example, the mechanisms and associations between work characteristics and changing functioning associated with retirement. With this point of view it would be possible to examine also the different developmental patterns in health functioning both before and after retirement. The question here would be which social and health-related or other factors contribute to improving or declining functioning.

## Limitations

This review included studies published during 2011 and 2021. This decision was made to gain as recent information as possible, however, most of the studies were based on cohort studies that drew on data collected during 1985 to 2014. Most of the studies included data from questionnaires completed after 2010. This review did not consider the differences between studies including “older” or “newer” data, but this could possibly provide interesting information itself.

Retirement policies among different countries vary a lot. The differences can be found, for example, in social policies and pension schemes. These differences might affect how functioning changes after retirement, and the heterogeneity in the review’s results. However, the strength in the review is that it considered only studies conducted in developed western countries. This might reduce the differences among countries, even though retirement policies among developed western countries also vary. This review did not consider participants’ health conditions pre and post retirement, which can strongly affect the functioning in general, since only 4 of the chosen studies used different diseases as covariates.

One aspect that potentially detracts from the trustworthiness of this review is that it was conducted by only 1 researcher. Consultation in scoping reviews is, however, optional from a methodology perspective.^16^ In this review, this was supplemented by utilising the opponents, supervisors, and study group comments along the review process, which improves the credibility and the dependability within the study.^21^

## Conclusions

The aim of this scoping review was to gain knowledge about a person’s functioning outcomes after retirement, to identify the studies available, and the possible knowledge gaps that exist in the field. The results of this scoping review showed that functioning changes in varying ways after retirement depending on the dimension of functioning. Generally, functioning can either improve or decline, and the same explanatory factors can influence both of these options. For example, retirement itself is associated with both declining and improving social functioning after retirement. What lies behind these associations varies; poor quality of life can follow retirement, but also the amount of leisure-time activities can increase. The results imply that further systematic review should possibly focus on 1 dimension of functioning, or studies using similar measures to gain a more coherent understanding of the phenomenon. This study also underlines the need for further research around the field to gain a more profound understanding about retirement at individual level, and to gain knowledge about the explanations behind the changes in functioning after retirement. For example, the mechanisms between work characteristics and changing functioning, which are associated with retirement, should be studied.

## Supplemental Material

sj-docx-1-inq-10.1177_00469580221142477 – Supplemental material for Functioning Changes in Varying Ways After Retirement: A Scoping Review Click here for additional data file. Supplemental material, sj-docx-1-inq-10.1177_00469580221142477 for Functioning Changes in Varying Ways After Retirement: A Scoping Review by Pauliina Saha, Jatta Salmela, Tea Lallukka and Anna Liisa Aho in INQUIRY: The Journal of Health Care Organization, Provision, and Financing
