# Supplementary material for: Functioning Changes in Varying Ways After Retirement: A Scoping Review
Source: Inquiry. 2023 Jan 5;60:00469580221142477. doi: 10.1177/00469580221142477 (PMC9830080; doi:10.1177/00469580221142477)
Supplement: sj-docx-1-inq-10.1177_00469580221142477 – Supplemental material for Functioning Changes in Varying Ways After Retirement: A Scoping Review [file sj-docx-1-inq-10.1177_00469580221142477.docx]

Figure 1 describes the literature search. The whole literature search process is clarified in the main text. The chosen keywords among databases varied to some degree because not all of the databases shared a similar range of keywords. However, all of the database searches included the search terms “retirement” and “functioning” cut with an asterisk. The CINAHL, MEDLINE and Medic databases did not have specific keywords for psychosocial, cognitive, physical, or social functioning – unlike the PubMed database. Instead, a subject heading “International Classification of Functioning, Disability, and Health” was found in all four databases. In PubMed, using the subject heading “International Classification of Functioning, Disability, and Health” did not broaden the results. Therefore, the subject heading was dropped from the literature search in the PubMed database. In Medic, the keyword “physical functional performance” was included to broaden the results since the amount of returned studies was small in general. This keyword was not found in CINAHL, MEDLINE or PubMed.  In CINAHL, MEDLINE, and PubMed, a search term “after” was used in its own search field for titles and abstracts to focus on the search for the review context. This term was used to target studies where the focus is on the population after retirement. This search term was not used in Medic since the amount of results was already relatively small.

Declining functioning included declining physical, social, cognitive, and mental functioning (Figure S1). Declining physical functioning was associated with age and work-related characteristics such as high job strain, passive work, and occupational environmental hazards. Declining physical functioning was also associated with health and retirement itself.

DECLINING

PHYSICAL

FUNCTIONING

- Age along with retirement declining physical functioning (25, 26, 34, 35)
- Declining physical functioning related with job features (26, 27, 32)
- Declining physical functioning related with workload (27, 28, 33, 36)
- Health causing declining physical functioning (34)
- Retirement declining physical functioning (25, 30, 34, 41)

DECLINING

SOCIAL

FUNCTIONING

- Declining social functioning related with lower cognitive level job (40)
- Retirement declining social functioning (41, 42)

DECLINING

COGNITIVE

FUNCTIONING

- Age declining cognitive functioning (28)
- Declining cognitive functioning related with socioeconomic position (23)
- Declining cognitive functioning after retirement related with high job strain (28, 33)
- Declining cognitive functioning related with health status (23)
- Declining cognitive functioning related with job features (22, 33)
- Declining cognitive functioning related with social isolation (31)
- Declining cognitive functioning related with workload (31, 33)
- Retirement declining cognitive functioning (22, 23, 30, 38)
- Retirement declining memory (23)

DECLINING

MENTAL

FUNCTIONING

- Age along with retirement declining mental functioning (29)
- Declining mental functioning related to social isolation (29)
- Retirement declining mental functioning (41)
- Retirement declining mental wellbeing (41)

DIMENSION OF FUNCTIONING

DRIVERS

Figure S1: Declining functioning characteristics. Source: Elaborated by the authors.

Several studies identified improving functioning after retirement. Improving functioning was found with physical, social, cognitive, and mental functioning (Figure S2).

|  |
| --- |

IMPROVING PHYSICAL

FUNCTIONING

- Increasing leisure-time activity (26)
- Preserved activities improving physical functioning (42)
- Retirement improving physical functioning (25, 30, 35, 40, 41)
- Self-rated health related with improving physical functioning (42)
- Socioeconomic position related with improving physical functioning (29)
- Workload related with improving physical functioning (27)

IMPROVING
SOCIAL

FUNCTIONING

- Favorable work related with improving social functioning (39)
- Increased autonomy improving social functioning (42)
- Perception of time improving social functioning (42)
- Retirement improving social functioning (30, 39, 42)

IMPROVING

COGNITIVE

FUNCTIONING

- Favorable work characteristics related with improving cognitive functioning (33, 39, 40)
- High job strain related with improving cognitive functioning (28, 33, 40)
- High work-related mental demands improving cognitive functioning (24, 31)
- Leisure-time activities improving cognitive functioning (39, 40)
- New leisure-time activities improving cognitive functioning (39)
- Retirement improving cognitive functioning (24)
- Retirement timing related with cognitive functioning (29, 40)
- Socioeconomic position related with improving cognitive functioning (23, 29, 40)

IMPROVING

MENTAL

FUNCTIONING

- Favorable work characteristics related with improving mental functioning (24)
- Health related with improving mental functioning (24)
- Retirement improving mental functioning (24, 30, 41)
- Sex related with improving mental functioning (24, 41)
- Socioeconomic position related with improving mental functioning (24, 29)

DIMENSION OF FUNCTIONING

DRIVERS

Figure S2: Improving functioning characteristics. Source: Elaborated by the authors.

Retirement was associated with inequalities in functioning, in terms of age, sex, marital status, race, socioeconomic position, and job features (Figure S3).

UNCOVERING INEQUALITIES IN COGNITIVE FUNCTIONING

- Revealing job features related inequalities in cognitive functioning (22, 24, 31)
- Revealing sex related differences in cognitive functioning (28)
- Revealing socioeconomic inequalities in cognitive functioning (38)

UNCOVERING INEQUALITIES IN PHYSICAL FUNCTIONING

- Revealing race related differences in physical functioning (35)
- Revealing retirement age related differences in physical functioning (35)
- Revealing sex related differences in physical functioning (32, 35)
- Revealing status related differences in physical functioning (35)
- Narrowing social class related differences (25)

EQUALISATION OF DIFFERENCES IN PHYSICAL FUNCTIONING

ISSUES IN FUNCTIONING

DRIVERS

Figure S3: Inequalities in functioning characteristics. Source: Elaborated by the authors.

Table S1: Table including chosen studies

| **Authors, year, country** | **Aim** | **Data & methods** | **Key results** | **Quality assesment** |
| --- | --- | --- | --- | --- |
| 22. Dawn C. Carr, Robert Willis, Ben Lennox Kail &  Laura L. Carstensen. 2020. USA. | To examine cognitive changes  as a function of job complexity in the context of different types of retirement transitions. | A quantitative, longitudinal study.  Data from the Health and retirement Study (HRS).  The data for the present analyses was drawn from the biannual waves 1996–2010. Sample consists of retired and full-time workers.  Age distribution from the age of 50.  n=2294 | All retirement pathways were associated with accelerated cognitive decline for workers in low complexity jobs. For high complexity workers retirement was not associated with accelerated  cognitive decline. | 10/11 |
| 23. Sean A. P. Clouston & Nicole Denier. 2017. USA | To determine whether there is evidence for a longitudinal relationship between retirement and cognitive aging, and if selective factors explain associations between  retirement and cognition. | A quantitative, longitudinal study.  Data from the Health and retirement Study (HRS).  The data for the present analyses was drawn from the biannual waves 1998–2012.  Age distribution from the age of 50.  n=18575 | Significant associations were found linking longer retirement with more rapid cognitive decline. | 11/11 |
| 24. Gwenith G. Fisher, Alicia Stachowski, Frank J. Infurna, Jessica D. Faul, James Grosch, Lois E. Tetrick. 2014. USA. | To examine whether cognitive job complexity or mental work demands are related to cognitive functioning in relation to retirement. | A quantitative, longitudinal study.  Data from the Health and retirement Study (HRS).  The data for the present analyses was drawn from the biannual waves 1992–2010.  Age distribution 50–70.  n=4182 | Working in an occupation characterized by higher levels of mental demands was associated with slower rate of cognitive decline after retirement. | 10/11 |
| 25. Eero Lahelma, Olli Pietiläinen, Tarani Chandola, Martin Hyde, Ossi Rahkonen & Tea Lallukka. 2019. Finland. | To examine whether transitions to retirement modified social class trajectories in physical functioning among Finnish female employees. | A quantitative, longitudinal study.  Data from the Helsinki Health Study (HHS).  The data for the present analyses were drawn from phases 2000–2002, 2007 and 2012.  Age distribution 40–72.  n=6976 | After mandatory retirement, functioning deteriorated in, whereas after disability retirement, functioning improved. Functioning converged at older ages.  Social class inequalities remained in all employment status groups. | 11/11 |
| 26. Jouni Lahti, Mikko Laaksonen, Eero Lahelma & Ossi Rahkonen. 2011. Finland. | The aim of this study was to investigate whether transition to old-age or disability retirement affects leisuretime physical activity. | A quantitative, longitudinal study.  Data from the Helsinki Health Study (HHS).  The data for the present analyses were drawn from phases 2000–2002 and 2007. Those who were on disability retirement at the follow-up were distinguished from oldage retirees.  Age distribution from the age of 40.  n=7332 | Among those transferred to old age  retirement moderate-intensity leisure-time physical activity increased,  and in addition, the occurrence of physical  inactivity reduced.  Such changes were not observed among those transferred to disability retirement. | 11/11 |
| 27. Minna Mänty, Anne Kouvonen, Tea Lallukka, Jouni Lahti, Eero Lahelma & Ossi Rahkonen. 2016. Finland. | To examine the association between pre-retirement physical working conditions and changes in physical health functioning during the retirement transition process. | A quantitative, longitudinal study.  Data from the Helsinki Health Study (HHS).  The data for the present analyses were drawn from phases 2000–2002 and 2012, focus on those who retired full time due to old age.  Age distribution from the age of 40.  n=1658 | Higher pre-retirement exposure to physical workload and environmental hazards was associated with lower physical health functioning after retirement.  The differences in functioning narrowed during the retirement transition process, as physical health functioning in the higher exposure groups improved significantly compared to lower exposure groups. | 11/11 |
| 28. Charlotta Nilsen, Monica E. Nelson, Ross Andel, Michael Crowe, Deborah Finkel & Nancy L. Pedersen. 2021. Sweden. | To examine associations between job strain and trajectories of change in cognitive functioning after retirement. | A quantitative, longitudinal study.  Data from the Swedish Adoption/Twin Study of Aging (SATSA).  Participants were followed for up to 27 years.  Age distribution from the age of 50.  n=307 | Greater job strain was associated with worse memory, speed, spatial ability, and general cognitive ability at retirement. It was also associated with slower decline in general cognitive ability, and slower decline in memory after retirement.  Post-retirement speed was not affected by job strain. | 10/11 |
| 29. Loretta G. Platts, Elizabeth Webb, Marie Zins, Marcel Goldberg & Gopalakrishnan Netuveli. 2015. United Kingdom. | To examine pathways linking social position in middle age to quality of life following retirement in French men and women. | A quantitative, longitudinal study.  Data from the GAZEL study.  The data combines detailed administrative data with  self-completion questionnaires, completed since 1989.  Age distribution 36–66.  n=11293 | Higher occupational grade in 1989 was associated with better quality of life 16 years later. This association was accounted for by individuals’ social status, mental health, physical functioning, and wealth. | 10/11 |
| 30. Beverly A. Roberts, Rebecca Fuhrer, Michael Marmot & Marcus Richards. 2011. United Kingdom. | To examine whether retirement is significantly associated with cognitive change after adjusting for preretirement cognitive function, personal, social, health and lifestyle factors, work characteristics and leisure activity. | A quantitative, longitudinal study.  Data from the Whitehall II study.  The data drawn from two phases resulting in an average of 5 years of follow-up between phases. Two groups were formed (retired, working).  Age distribution from the age of 35.  n=2031 | Retirees were involved in more leisure activities than those in  employment.  Current  self-rated health was the same in both groups.  Mental health and relative self-rated physical health were better in retirees. The retired group had signiﬁcantly higher frequencies of angina and hypertension diagnoses. | 11/11 |
| 31. Francisca S. Rodriguez, Matthias L. Schroeter, A. Veronica Witte, Christoph Engel, Markus Löffler, Joachim Thiery, Arno Villringer, Tobias Luck & Steffi G. Riedel-Heller. 2016. United Kingdom. | To study whether high mental demands at work, which have shown to promote a good cognitive functioning in old age, could offset the adverse association between social isolation and cognitive functioning. | A quantitative, longitudinal study.  Data from the LIFE-Adult-Study.  Sample from LIFE data was combined with a subsample of 400 separately recruited individuals.  Age 40–79, subsample 18–39.  n=9173 | Cognitive functioning was significantly lower in socially isolated individuals and in individuals working in low mental demands jobs after retirement and even after considering the educational level.  An interaction effect suggested stronger effects of mental demands at work in socially isolated than non-isolated individuals. | 10/11 |
| 32. Erika L. Sabbath, M M. Glymour, Alexis Descatha, Annette Leclerc, Marie Zins, Marcel Goldberg and Lisa F. Berkman. 2013. United Kingdom. | To investigate whether health effects of combined occupational exposures during working life are observed after individuals retire and are no longer exposed. | A quantitative, longitudinal study.  Data from the GAZEL study.  Sample consisted of individuals retired 1995–2006.  n=9168 | Both psychosocial and biomechanical exposures during working life were independent predictors of the physical functioning (PF), physical difficulties (RL) and bodily pain (BP). | 10/11 |
| 33. Erika L. Sabbath, Ross Andel, Marie Zins, Marcel Goldberg & Claudine Berr. 2016. United Kingdom. | To test whether exposure to job demands, job control and their combination during working life predicted post-retirement performance on eight cognitive tests. | A quantitative, longitudinal study.  Data from the GAZEL study.  The sample consisted of cohort members who had undergone post-retirement cognitive testing.  Age distribution from the age of 55.  n=2149 | Low job control during working life was negatively associated with executive function, psychomotor speed, phonemic fluency and semantic fluency after retirement.  Both passive and high-strain jobs were associated with lower scores on phonemic and semantic fluency when compared to low-strain jobs. | 9/11 |
| 34. Sari Stenholm, Hugo Westerlund, Paula Salo, Martin Hyde, Jaana Pentti, Jenny Head, Mika Kivimäki & Jussi Vahtera. 2014. Finland. | To examine the age-related trajectories of physical functioning among those in full-time work and retirement. | A quantitative, longitudinal study.  Data from the Health and retirement Study (HRS).  Sample consists of participants who were working full-time or were in full-time retirement and 65-85 years of age during the follow-up period 1992–2010.  Age distribution from the age of 50.  n=17844 | The number of physical functioning difficulties increased every 10 years more steeply among those in retirement compared to those in full-time work. | 11/11 |
| 35. Sander K. R. van Zon, Ute Bültmann, Sijmen A. Reijneveld & Carlos F. Mendes de Leon. 2016. USA. | To examine the pattern of pre- and post-retirement changes in functional health and to examine the degree to which socioeconomic position (SEP) modifies pre- and post-retirement changes in functional health. | A quantitative, longitudinal study.  Data from the Health and retirement Study (HRS).  Sample consisted of those with at least one measurement wave prior to, and one after they reported retirement.  Age distribution 50–70.  n=7242 | Average levels of limitations in mobility and large muscle functions increased significantly in the years prior to retirement. This increase slowed down after retirement, most prominently for limitations in large muscle functions.  Higher SEP was associated with a slower increase of functional limitations prior to retirement. After retirement, a less clear pattern was found as only wealth modified the increase of limitations in mobility functions. | 10/11 |
| 36. Morten Wahrendorf, Grace Sembajwe, Marie Zins, Lisa Berkman, Marcel Goldberg & Johannes Siegrist. 2012. USA. | To study long-term effects of psychosocial work stress in mid-life on health functioning after labor market exit. | A quantitative, longitudinal study.  Data from the GAZEL study.  The sample consisted of individuals employed 1997–1999 and who were retired in 2007.  n=6053 | Consistent effects of both work stress models and their single components on mental and physical health functioning during retirement were observed. | 10/11 |
| 37. Kandauda (K.A.S.) Wickrama, Catherine Walker O’ Neal. 2013. USA. | To examine the influence of work status on subsequent memory trajectories in later years. | A quantitative, longitudinal study.  Data from the Health and retirement Study (HRS).  Study used a subsample of older adults who were 65 years or older in 2002, sample categorized (full-time working, fully retired, partially retired not worked).  Age distribution from the age of 65.  n=8165 | Fully retired individuals,  those who did not work, and those working part-time showed greater deterioration in their immediate memory.  The findings from these growth curve analyses support the hypothesis that working late in life helps to impede immediate memory declines that often occur in later years. | 10/11 |
| 38. Baowen Xue, Dorina Cadar, Maria Fleischmann, Stephen Stansfeld, Ewan Carr, Mika Kivimäki, Anne McMunn and Jenny Head. 2018. Netherlands. | To study whether a lack of mentally challenging activities might exacerbate the loss of  cognitive function. | A quantitative, longitudinal study.  Data from the Whitehall II study.  The data for the present analyses were drawn from phases 1997–1999, 2002–2004, 2007–2009 and 2012–2013.  Age distribution 45–83.  n=3433 | All domains of cognition declined over time. Declines in verbal memory were 38% faster after retirement compared to before.  The rate of post-retirement verbal memory decline was similar across different employment grades. | 10/11 |
| 39. Catherine Grotz, Fanny Matharanb, Hélenè Amievab, Karine Pérès, Sonia Laberon, Anne-Marie Vonthron,  Jean-Francois Dartigues, Stéphane Adam, & Luc Letenneur. 2016. France. | To investigate the relationship between psychological transition and adjustment to retirement and cognitive performances in older adults. | A quantitative study, cross sectional study.  Data from the Approche Multidisciplinaire Intégrée study.  The data for the present analyses was drawn from the baseline study.  Age distribution from the age of 65.  n=590 | Positive consideration of former work situation, development of new activities during retirement and good adaptation to free time – were associated with better cognitive performances. | 7/8 |
| 40. Andreas Ihle, Catherine Grotz, Stéphane Adam, Michel Oris,  Delphine Fagot, Rainer Gabriel & Matthias Kliegel. 2016. United Kingdom. | To investigate the association of timing of retirement with cognitive performance. | A quantitative study, cross sectional study.  Data from the Vivre-Leben-Vivere (VLV) survey.  The main cross-sectional data for the present analyses were collected in 2011 and 2012.  Age distribution 65–101.  n=2263 | Earlier retirement was significantly associated with better performance in the used tests.  Individuals with a moderate number of leisure activities in old age, earlier retirement was related to better cognitive performance, but not in those with a relatively large number of leisure activities. | 7/8 |
| 41. Elżbieta Biernat, Łukasz Skrok & Justyna Krzepota. 2019. United Kingdom. | To assess how retirement affects the short-term and medium-term sport/exercise activity (S/EA) of Poles. | A quantitative, quasi-experimental study.  Data from the  Social Diagnosis study.  The data for the present analyses were drawn from phases 2011, 2013 and 2015. Being retired in 2013 was used as a treatment variable.  Age distribution from the age of 50.  n=5610 | Retirement does not have a significant effect in the short term on S/EA of men and women.  In the medium perspective, retirement had the effect on reducing S/EA among men and various dimensions of their social life. | 6/9 |
| 42. Lars Bauger & Rob Bongaardt. 2016.Norway. | To identify and describe the general meaning structure of the experience of well-being after retirement. | Phenomenological study.  Retirees (n = 9) were interviewed, age range 62–71.  Data analysed with  Giorgi’s descriptive phenomenological method. | A new experience of time presents possibilities for action.  The increased sense of autonomy solicited the agency of the retiree. | 8/10 |
